# Predictive Molecular Tumour Testing: What Are the Obstacles between Bench and Bedside?

**DOI:** 10.1155/2012/838509

**Published:** 2012-05-14

**Authors:** Linda Mileshkin, Bhaumik Shah, Michael Michael

**Affiliations:** Division of Cancer Medicine, Peter MacCallum Cancer Centre, St. Andrews Place, East Melbourne, VIC 3002, Australia

## Abstract

There have been many exciting new breakthroughs in understanding tumour biology. This has opened up the possibility of personalized treatment for people with certain tumours. The epidermal growth factor receptor (EGFR) and K-ras are two such targets that can help classify tumours on a molecular basis and guide treatment decisions. However, there are still questions about how best to implement new molecular tests like these to characterize tumours in clinical practice. Potential obstacles include availability of good quality tissue specimens, access to the right test, and consensus about interpretation, funding, and availability. In this paper, we review these issues, by discussing these two examples in detail and suggest some actions for addressing potential barriers.

## 1. Introduction

The landscape of treatment options in medical oncology is changing rapidly. Apart from traditional cytotoxic medications, there are now increasing numbers of targeted biological agents and immunotherapy treatments. We are developing better insights into the molecular biology of neoplastic disease and the pharmacology/pharmacodynamic of cytotoxics and novel biologic agents. However, there are vast areas of uncertainties about efficacy and toxicity prediction of various treatments. There are multiple factors related to the tumour and the patient that play crucial role determining these properties. Molecular testing of tumours is a field that is starting to shed light on this area and permits us to make more individualised treatment decisions, including choosing the right drug for the right patient.

There are many specific molecular tests that are already in routine clinical use such as testing HER-2 in breast cancer, bcr-abl in CML, and c-Kit in gastrointestinal stromal tumours. There are other tests which have sufficient evidence for clinical utility but are not yet widely available or government funded. Here we explore the issues that lead to delay in routine implementation of these kinds of tests by discussing the examples of K-ras mutation testing in colon cancer and EGFR mutation testing in lung cancer.

## 2. K-ras

Cetuximab and panitumumab are anti-EGFR monoclonal antibodies that are usually considered in the 2nd to 3rd line treatment setting for metastatic colorectal cancer and in selected patients for first-line therapy where downstaging prior to curative metastasectomy is being considered. There are downstream components from EGFR that can determine the efficacy of this strategy. K-ras, short for Kirsten ras, is one such downstream G protein. Its mutation can confer stimulus-independent activation of the pathway. The prevalence of the mutation in colorectal cancer is 30–60% and the mutation status in the primary tumour and metastasis from the same individual is reported to be highly concordant [1–5].

The presence of a K-ras mutation in a tumour is now widely accepted as a negative predictive factor for response to anti-EGFR antibody treatment. Several studies have shown lack of any benefit or actual detriment from anti-EGFR antibody treatment in colorectal cancer patients if K-ras is mutated [6–13]. For example, as a single agent in the setting of advanced colorectal cancer, Cetuximab results in an improvement in median survival of around 4.7 months (near doubling) compared to placebo in patients with K-ras wild-type (WT) tumours but no statistically significant difference in patients with K-ras mutant tumours [6]. In quantitative terms, 0.39 life years (LY) and 0.25 quality adjusted life years (QALY) are gained.

In several countries Cetuximab treatment is government funded only for patients with K-ras wild-type colorectal cancer, in whom it has been determined to be cost-effective treatment. Hence testing for the presence of a K-ras mutation is considered a codependent technology. However the testing is not currently funded by public or private payers in many places such as Australia. It can be accessed by a patient self-funding the test, the pharmaceutical company's special access program or as part of a clinical trial. Various questions about testing that have been obstacles to government funding of testing and potential answers are discussed below.

### 2.1. When to Test?

A K-ras mutation test can potentially be requested at different time points in the natural history of colorectal cancer. This could be at (a) initial diagnosis of colorectal cancer, (b) at diagnosis of metastasis, or (c) at failure of 1st-line treatment for metastatic disease.

The decision requires consideration of cost, turnaround time, and difficulties in retrieval of adequate tissue for testing. Option (a) is the simplest but the most costly. It allows adequate good quality tissue to be available for most of the patients. However, a large number of patients with locally advanced CRC will not progress to metastatic disease and hence the result of K-ras testing wouldn not be needed as it is not in itself a prognostic marker. Option (c) is the cheapest as it would test only those patients who need the result. However, it falls down due to potential time lost in retrieval and testing of a specimen when the window of opportunity for treatment may be short.

Option (b) is a fair compromise between the two as it will allow the result to be available in a timely manner for patients before a decision needs to be made about second-line treatment for most patients. Nevertheless it may still result in small numbers of patients being tested who die before 2nd-line treatment can be initiated or may be cured by first line therapy including surgery for oligometastatic disease.

### 2.2. What to Test?

In some settings the presence of somatic mutations in tumours can vary between the primary and metastases. Fortunately, the K-ras mutation status is thought to be stable between a colorectal tumour primary and metastatic deposit, with the correlation between primary and metastases reported to be 93% (range 76–100%) [14–16]. This means that the test can usually be done on the primary resection specimen, even though the result is used for the treatment of metastatic disease which may not develop until some years down the track. However, if enough good quality tissue is not available, then biopsy of a site of metastatic disease may be required.

There are several sources of false-positive and false-negative results for K-ras testing of tumour samples, with obvious flow on effects including the denial of a useful treatment or use of inappropriate therapy, respectively. For example, formalin fixed paraffin embedded (FFPE) specimens can have artificial mutations induced during the processing resulting in reduced specificity. However, if there is enough cancer cells in a specimen than this random error can be avoided. Reactive cells in the tissue stroma and neighbouring normal cells can also compete for DNA amplification with cancer cells and can reduce sensitivity of the test [17]. Macro-or micro-dissection or taking a selective needle core from a tissue block are ways to enrich the specimen to be tested for tumour. Care must be taken to obtain enough tumour tissue to have enough DNA for testing. At least 300 cancer cells or 30 ng of DNA is reported to optimize yield [18].

### 2.3. How to Test?

There are a multitude of available methods to detect K-ras mutations. International recommendations are that a sensitive, specific, and reliable DNA-based assay be used [19]. The common methods are direct sequencing, RFLP (Restriction Fragmentation Length Polymorphism), ASO (Allele Specific Oligonucleotide) Hybridization, HRMA (High Resolution Melting Analysis), ARMS (Amplification Refractory Mutation System), and pyrosequencing. There is no common test that has been used across multiple clinical trials. There are drawbacks for all methods with limitations in sensitivity, specificity, or practicability due to cost or turnaround time [17]. Nevertheless, there is still reasonable concordance between results by the different testing methods [20].

Direct sequencing is considered the gold standard as it is able to detect all mutations in the K-ras gene but also has some drawbacks. For example, direct sequencing requires a minimum 20% mutation load (mutant cells/total cells) to be positive. Some nonmutant tumours (as per direct sequencing) turned out to harbour mutant cells when examined with the more sensitive pyrosequencing method [21]. In another study, direct sequencing was compared directly with mutation-enriched sequencing in 90 colorectal carcinomas. K-ras mutations were detected in 40% by means of direct sequencing and 55% by means of mutation-enriched sequencing. Hence minor clones were detected in about 15% of colorectal cancers [22].

The essential requirement is that the testing is validated and performed in an accredited laboratory with an external quality assurance program. The availability of the test is not widespread so this may require transport of appropriate tissue, once it is identified and retrieved from storage, from the original laboratory to another laboratory. This adds further delay in getting results. It is helpful when requesting tissue from the source laboratory to indicate how urgent or not the request is for patient care.

### 2.4. How to Interpret the Result of Testing?

Generally if a K-ras mutation is detected then a patient will not be considered appropriate for anti-EGFR antibody treatment. The use of highly sensitive detection methods will in more mutations be identified [23]. However, the threshold mutation load within a K-ras mutant tumour specimen is not well defined; beyond which there is definite futility of the use of anti-EGFR antibodies in patients. For example, treatment response has been described in tumours which harbour only a small percentage of mutated cells (<20%) [21]. Hence caution has been recommended in the interpretation of the use of very sensitive testing methods such as pyrosequencing. The threshold used in the relevant clinical trials has unfortunately not been consistently reported as outlined in Table 1 [6].

The known mutations are few. Ninety percent involve 3 codons (Exon 2: codon 12–79%, codon 13–17.6%, and Exon 3: codon 61–1%) [17]. It is not yet clear if the biology of different mutations is similar. There are suggestions that certain mutations (e.g., G13D) do not confer resistance to Cetuximab, though these observations need to be confirmed by prospective trials and were not replicated in analysis of a recent panitumumab trial [26, 27]. Currently, it is not routine to differentiate specific mutations for clinical decision making.

### 2.5. Is Testing Sufficient to Predict Response?

K-ras wild-type status is necessary but not sufficient to predict response to treatment. Although it is clear that the vast majority of patients with a K-ras mutant tumour will not respond to treatment, nearly 60% of patients will also not have a response to anti-EGFR antibody treatment despite having a K-ras WT tumour. Other potential predictive markers have also been post hoc or retrospectively identified such as EGFR gene copy number, mRNA expression for the EGFR ligands, epiregulin and amphiregulin, and wild-type BRAF [11, 28, 29]. There will most likely be others identified with time; however in all cases confirmation is required in well-designed prospective trials.

### 2.6. Is Testing Cost-Effective?

It is established that use of anti-EGFR antibodies such as cetuximab is only cost-effective if used for the treatment of patients with WT K-ras tumours [30]. Most of the pivotal clinical trials have focussed on efficacy and safety and some have not included prospective economic evaluation of the therapeutic intervention. Previously it has not been routine to include economic analysis of codependent diagnostic tests in clinical trials. However this is needed before policy makers allocate funds for testing in routine clinical practice.

Determining the true cost of testing is not straightforward. Whether you include only direct costs (salary for staff, reagents, and equipment) or indirect costs as well (tissue retrieval from another institution) generates a substantial difference in costing. One economic modelling proposed by Merck Sorano to the Australian Pharmaceutical Benefits Advisory Committee showed a cost of AU$45000–75000 for use of Cetuximab, including K-ras testing, per QALY gained. This was considered high but still reasonable and eventually resulting in the government approving the funding of Cetuximab. The presumed cost of the test itself in the model made up only a very small component of the cost at AUD 250 and is suggested to be cost-effective based on recent modelling from a Swiss group [31]. However, currently the test is not funded by the Australian government as it has yet to get through the approval pathway for codiagnostic testing which has historically been a separate and disjointed process from the drug approval pathway. The regulatory authorities are now attempting to streamline the process by concurrent evaluation of the diagnostic tests with drug approval.

## 3. EGFR

EGFR tyrosine kinase inhibitors (TKIs) are known to result in dramatic responses and prolonged survival times for patients with EGFR mutant non-small-cell lung cancer (NSCLC). They are used in the 1st- to 3rd-line treatment setting in patients with EGFR activating mutations as well as maintenance treatment after first-line chemotherapy. It has been recently established that EGFR testing is essential before giving an EGFR TKI in the first-line setting as there is negative impact of giving an EGFR TKI rather than chemotherapy to a patient who is EGFR mutation negative [32]. Studies have found that approximately 10% to 20% of NSCLC tumours harbour somatic mutations in the EGFR gene [33, 34].

In Australia treatment with Gefitinib is funded for the treatment of relapsed NSCLC after chemotherapy, but only for EGFR mutant patients. Erlotinib is also available for the treatment of patients who relapse after chemotherapy, but its use is not restricted by EGFR status because of the BR21 trial which demonstrated benefit from use in this setting in patients who were not prospectively tested for mutations [35]. First-line treatment is not currently funded.

Also, testing for EGFR mutations is not currently government funded although like K-RAS testing it is considered a codependent technology for first-line treatment. It is available either as part of various clinical trials, in house funding by some public hospitals, an access program run by a pharmaceutical company, or the patient paying out-of-pocket for the test.

### 3.1. When to Test?

The test result is relevant for any patient and their doctor considering treatment of metastatic NSCLC. The chances of finding an EGFR mutation are significantly higher in patients who are never or light smokers, or those who have nonsquamous histology [36, 37]. Hence it is proposed that all such patients should have testing for EGFR mutations. Patient who continue to smoke do not appear to benefit from treatment with an EGFR TKI due to effects on drug metabolism; therefore testing may not be of relevance unless the patient is willing to try to stop smoking [38]. Given that a test result may take many weeks to obtain, it is also sensible to test patients at diagnosis if they have locally advanced disease which may be potentially curable but where the chance of relapse is high, for example, stage III disease. It has also been suggested that there could be a time and cost saving by having the original reporting pathologist arranging for the test to be performed while actively reporting the case rather than having it retrieved for testing later.

One suggestion for selecting an enriched subset to test is to use epidemiologic factors. The typical epidemiologic factors associated with higher rates of EGFR mutation are adenocarcinoma histology, female sex, never-smoker status, and eastern Asian ethnicity. But using these features to narrow down the patient subgroup for testing can miss some patients as the mutation has been found, though in lower frequency, in other subsets too (smokers, men, and other ethnicities) [39]. It has been suggested that 31% of all EGFR mutations would be missed if testing was restricted to women, 40% would be missed if testing was restricted to never smokers, and 57% would be missed if testing was restricted to women who never smoked cigarettes. The mutation is very rare in squamous histology (0–1.1%) and hence the utility of testing these patients is low [40, 41].

### 3.2. What to Test?

Testing for EGFR mutation status is usually performed on the primary specimen that led to a diagnosis of non-small-cell lung cancer as this may be the only available tissue. This is a reasonable and practical approach. However, there are reports suggesting potential discordance between the primary tumour and metastases regarding EGFR status [42]. Tumour progression and treatment can potentially change the mutation status and create discrepancy between the primary and metastasis. Hence treatment decisions based purely on histology from the primary tumour can be misleading when the focus of treatment is on metastasis. Even within same tumour there may be quite a lot of heterogeneity in terms of the presence and type of mutation [43].

Nowadays, due to reliance on minimally invasive techniques for diagnosis such as transbronchial or image-guided percutaneous needle biopsies, sometimes available diagnostic material may be limited. Generally a core biopsy at a minimum is required for EGFR testing. Hence having cytology alone for diagnosis is not currently recommended although ways to test for mutations in cytological specimens and/or blood are being investigated. Poor quality tissue with low tumour/surrounding tissue ratio (<20%) can lead to false-negative mutation testing results. To avoid this proper planning for biopsy is needed. Sometimes PET guidance to help target the best tissue for biopsy may be useful.

### 3.3. How to Test?

The finding that is most convincingly associated with response to EGFR TKI treatment is the presence of an EGFR mutation in NSCLC. Some studies also report a correlation between treatment responses and positive immunohistochemistry or FISH testing for EGFR expression. However this association is much less robust and not found in all studies and hence not recommended as a way to select patients for treatment.

A number of different methods for detection of an EGFR mutation can be used such as DNA sequencing, HRMA, or ARMS. There is no single agreed-upon method; however direct sequencing is currently considered the gold standard as it can detect any mutation in the EGFR [34]. As with K-ras testing the sensitivity and specificity of the test may be affected by tissue handling and fixation issues. Macro- or micro-dissection of a tumour block may help to increase the chance of successfully detecting a mutation as generally a biopsy needs to contain at least 20% tumour cells to provide a reliable sequencing result [33].

Currently available testing facilities for direct sequencing are limited. For example, only 5 laboratories in Australia perform the test as of 2010 with no external quality assurance program. This makes equitable access to routine testing of mutation status difficult.

### 3.4. How to Interpret the Result of Testing?

There is strong evidence for considering EGFR mutation as a good predictive marker for response to an EGFR TKI and it is appropriate for these patients to receive treatment with an EGFR TKI for the treatment of metastatic disease [32]. Ideally this would be given as first-line treatment if accessing a test result in a timely fashion and funded treatment were available. However, it appears that as long as patients with EGFR mutations receive an EGFR TKI at some stage in their treatment, either before or after chemotherapy that overall survival is similar. However, as it is known that a significant percentage of patients are too unwell to consider second line therapy, then first-line treatment with an EGFR TKI is preferable. This is also the case for more frail patients who may not be able to tolerate chemotherapy treatment but could benefit significantly from an EGFR TKI.

Ninety percent of sensitizing mutations involve codon 19 (in-frame deletion or insertion mutations) or codon 21 (L858R missense mutation). Other mutations in the EGFR may be associated with treatment resistance or are of uncertain significance (neutral), for example, exon 18 deletion. Secondary mutations in the EGFR such as T790M are described to be associated with resistance to EGFR TKIs [44]. It is important that the treating oncologist takes note of the exact type of EGFR mutation identified and ensures that it is the one described to be associated with response to an EGFR TKI before starting therapy.

Alternatively, not every patient requires an EGFR mutation to get benefit from an EGFR TKI. As described above the BR21 study determined that Erlotinib can be of benefit for the treatment of relapsed disease after prior platinum-based chemotherapy in NSCLC patients regardless of mutation status. Also according to the result of SATURN study, Erlotinib can be given as maintenance treatment after 1st-line chemotherapy without knowledge of the EGFR mutation status and can still confer a small survival benefit [45]. However, analysis of the subset of patients in the trial who had tissue available for testing suggests that the benefit was primarily driven by those who did have EGFR mutant tumours.

### 3.5. Is Testing Sufficient to Predict Response?

Not every patient with EGFR mutant NSCLC will respond to an EGFR TKI. Similar to the story of EGFR monoclonal antibodies in colon cancer treatment, K-ras mutation can nullify the effect of a TKI. It has been debated if EGFR and K-ras mutations are mutually exclusive. But there are definite reports of cases with coexistence of the both mutations. However, in general patients with K-ras mutations will not respond to an EGFR TKI. PTEN loss or AKT activation by other mechanisms may also confer resistance to an EGFR TKI [46].

### 3.6. Is the Testing Cost-Effective?

The cost-effectiveness of EGFR mutation testing specifically has not been prospectively assessed within all of the pivotal clinical trials performed so far. Economic analysis of the BR21 trial suggested that with an incremental cost-effectiveness ration of $94,638 per life year gained, Erlotinib treatment for patients with previously treated advanced NSCLC is marginally cost-effective in an unselected patient population. The authors suggest that the use of molecular predictors of benefit for targeted agents could help to identify more or less cost-effective subgroups for treatment [47].

The cost-effectiveness of first-line treatment is still being evaluated. A recent assessment by an evidence review group for the National Institute of Health and Clinical Excellence (NICE) suggested that first-line treatment with Gefitinib may not be cost-effective at what would usually be considered standard levels of willingness to pay for an additional QALY, largely because of the cost of the drug treatment [48]. Of note the proposed cost of the test in Australia is only AUD $400–606. However, recent modelling from a Singapore group suggested that EGFR testing and first-line treatment with Gefitinib would be cost-effective compared to standard care, primarily because of the cost saving associated with not providing the drug to those who are not likely to benefit [49].

## 4. Conclusion

As we enter a new era of improved survival rates following a diagnosis of cancer, it is anticipated that targeted therapies will play an increasing role in treatment strategies. It is important that the development of predictive molecular testing of tumours occurs in parallel with the development of these new therapies in order to help select the best treatments for patients. In order for this to successfully occur it is necessary to have access to good quality tumour tissue for testing that can occur in a timely fashion, even if this needed some time after the tissue is acquired.

It is crucial that we have enough numbers of appropriate staffed laboratories to provide high-quality services for molecular tumour testing. It is also important that there is a framework to ensure quality control. The testing facilities need to be suitably resourced to be able keep pace with the new exciting tumour targets and treatments that are being discovered and shown to be promising in clinical trials, such as the recently developed tyrosine kinase inhibitors for BRAF mutations in melanoma and EML4-ALK mutations in NSCLC.

In addition, future clinical trials of targeted therapies need to be designed not only to assess the efficacy and cost-effectiveness of promising new drugs but also to assess the associated codiagnostic technologies in order to allow assessment by funding agencies and early integration in clinical practice. Education of the oncology workforce about the effective use of codiagnostics in patient management is also essential.

All of these requirements need to have a backing from policy makers and government funding bodies. Codependent technologies need to be able to be assessed by a transparent process simultaneously with new therapies to ensure rapid approval of both technologies and thus provide individualised and quality use of medicines for people affected by cancer.

## Figures and Tables

**Table 1:** Types of K-ras mutation performed in the pivotal clinical trials.

Author	Publication	K-ras mutation analysis	Validation and sensitivity
Peeters et al. [24]	Randomized Phase III Study of Panitumumab with Fluorouracil, Leucovorin, and Irinotecan (FOLFIRI) Compared with FOLFIRI Alone as Second-Line Treatment in Patients with Metastatic Colorectal Cancer	Blinded central laboratory using a validated K-ras mutation allele-specific PCR kit (DxS Ltd, Manchester, UK)^1^	Validated sensitivity cutoffs not published
Amado et al. [12]	Wild-type KRAS Is Required for Panitumumab Efficacy in Patients with Metastatic Colorectal Cancer	Blinded central laboratory using a validated K-ras mutation allele-specific PCR kit (DxS Ltd, Manchester, UK)^1^	Validated sensitivity cutoffs not published
Karapetis et al. [6]	K-ras Mutations and Benefit from Cetuximab in Advanced Colorectal Cancer	PCR K-ras analyses for exon 2 mutations in the Department of Clinical Biomarkers-Oncology at Bristol-Myers Squibb, Hopewell, N.J.	Validated sensitivity cutoffs not published
Van Cutsem et al. [25]	Cetuximab and Chemotherapy as Initial Treatment for Metastatic Colorectal Cancer	K-ras mutations in codons 12 and 13 with the use of a polymerase-chain-reaction (PCR) clamping and melting curve method	Sensitivity and validation published^2^
Douillard et al. [5]	Randomized, Phase III Trial of Panitumumab with Infusional Fluorouracil, Leucovorin, and Oxaliplatin (FOLFOX4) versus FOLFOX4 Alone as First-Line Treatment in Patients with Previously Untreated Metastatic Colorectal Cancer	K-ras testing as per allele-specific polymerase chain reaction (DxS, Manchester, United Kingdom)	Sensitivity and validation published^3^

(1) Identifies seven somatic mutations located in codons 12 and 13 (Gly12Asp, Gly12Ala, Gly12Val, Gly12Ser, Gly12Arg, Gly12Cys, and Gly13Asp).

(2) All relevant codon 12/13 K-ras mutations previously described detected. Sensitivity confirmed using a dilution series (up to 500-fold excess of K-ras wild-type DNA) of DNA derived from K-ras *mutant and wild-type cell lines *and on clinical FFPE CRC biopsies with tumor cell content <5%. Validated against specific cell lines with defined wild-type or K-ras mutations, and comparative analysis, using an alternative technique (DxS K-ras Mutation Detection Kit, DxS Ltd, Manchester, UK,)).

(3) External laboratory validated the assay for analytic/diagnostic performance, established acceptance criteria, and performed the K-ras analysis in a blinded fashion. Sensitivity parameters not published.
